# LC/MS-Based Untargeted Lipidomics Reveals Lipid Signatures of Sarcopenia

**DOI:** 10.3390/ijms25168793

**Published:** 2024-08-13

**Authors:** Qianwen Yang, Zhiwei Zhang, Panpan He, Xueqian Mao, Xueyi Jing, Ying Hu, Lipeng Jing

**Affiliations:** Institute of Epidemiology and Statistics, School of Public Health, Lanzhou University, Lanzhou 730000, China; yqw18709186692@163.com (Q.Y.); zzw982022@163.com (Z.Z.); hepanpan0518@163.com (P.H.); maoxueqian2023@163.com (X.M.); 18862232163@163.com (X.J.); 15822747125@163.com (Y.H.)

**Keywords:** untargeted lipidomics, LC/MS, lipid signatures, sarcopenia

## Abstract

Sarcopenia, a multifactorial systemic disorder, has attracted extensive attention, yet its pathogenesis is not fully understood, partly due to limited research on the relationship between lipid metabolism abnormalities and sarcopenia. Lipidomics offers the possibility to explore this relationship. Our research utilized LC/MS-based nontargeted lipidomics to investigate the lipid profile changes as-sociated with sarcopenia, aiming to enhance understanding of its underlying mechanisms. The study included 40 sarcopenia patients and 40 control subjects matched 1:1 by sex and age. Plasma lipids were detected and quantified, with differential lipids identified through univariate and mul-tivariate statistical analyses. A weighted correlation network analysis (WGCNA) and MetaboAna-lyst were used to identify lipid modules related to the clinical traits of sarcopenia patients and to conduct pathway analysis, respectively. A total of 34 lipid subclasses and 1446 lipid molecules were detected. Orthogonal partial least squares discriminant analysis (OPLS-DA) identified 80 differen-tial lipid molecules, including 38 phospholipids. Network analysis revealed that the brown module (encompassing phosphatidylglycerol (PG) lipids) and the yellow module (containing phosphati-dylcholine (PC), phosphatidylserine (PS), and sphingomyelin (SM) lipids) were closely associated with the clinical traits such as maximum grip strength and skeletal muscle mass (SMI). Pathway analysis highlighted the potential role of the glycerophospholipid metabolic pathway in lipid me-tabolism within the context of sarcopenia. These findings suggest a correlation between sarcopenia and lipid metabolism disturbances, providing valuable insights into the disease’s underlying mechanisms and indicating potential avenues for further investigation.

## 1. Introduction

Sarcopenia is a multifactorial disease commonly associated with aging, weakness, and cachexia [1]. Initially, in 2010, the European Working Group on Sarcopenia in Older People (EWGSOP) published a widely used definition of sarcopenia that emphasized the identification and care of individuals at risk for or with sarcopenia [2]. This definition has significantly advanced our understanding and management of the condition. In 2019, the Working Group met again (EWGSOP2) and revised the definition, which redefined sarcopenia as a progressive and systemic skeletal muscle disorder. It prioritizes low muscle strength as the primary indicator of probable sarcopenia, underscoring rapid losses in muscle mass and function, which increase the risks of falls, functional decline, weakness, and mortality [3]. However, owing to the multifaceted nature of the influencing factors and intricate outcomes involved, research elucidating the pathogenesis of this disease is currently lacking.

Sarcopenia primarily manifests as reduced muscle mass and muscle function (including muscle strength), diminished exercise capacity, physical dysfunction, and decreased physical fitness [4]. Previous studies have demonstrated that older adult individuals with sarcopenia exhibit distinct characteristics in terms of skeletal muscle phosphodiesters, phosphocreatine, and phospholipids [5]. As aging progresses, lipid droplets inside muscle cells gradually accumulate, facilitating fat infiltration and the buildup of free fatty acids, which inversely affects muscle strength [6]. The possible mechanisms proposed by the existing research mainly include the idea that intramyocellular lipid deposition leads to a decrease in mitochondrial numbers and increased production of reactive oxygen species, potentially compromising muscle function [7]; Specific fatty acids trigger the release of signaling molecules such as prostaglandins and leukotrienes, which induce skeletal muscle inflammation by involving the recruitment of inflammatory cells, muscle fiber necrosis, and edema. This further leads to muscle atrophy and impaired muscle performance [8]; Additionally, an abnormal lipid metabolism may induce insulin resistance and alter blood glucose levels in muscle cells, subsequently impairing the development, growth, and repair of skeletal muscles [9]. What is more, some pro-inflammatory lipids release paracrine hormones and cytokines that further encourage this lipid accumulation, creating a self-reinforcing cycle. This lipotoxicity reduces muscle fiber contractility and disrupts the synthesis of muscle proteins, thereby intensifying sarcopenia, illustrating how lipids may contribute to the development of this condition [10]. Previous mechanistic studies have primarily been conducted through animal experiments, focusing mainly on exploring the correlation between abnormalities in lipid metabolism and muscle function or quality. However, direct evidence in human sarcopenia is notably sparse. Some research have found that waist circumference and body mass index (BMI) were significantly related to reductions in physical function and muscle mass. Individuals with a larger waist circumferences and relatively higher BMI were more likely to have a significantly lower physical function [11]. However, a cross-sectional study showed that within the normal reference range, an increase in the lipid metabolism-related parameters BMI, triglyceride (TG), and total cholesterol (TC) may have a protective effect on sarcopenia [12]. Although these findings suggest that an abnormal lipid metabolism may be closely related to the occurrence of sarcopenia in populations, they do not clarify specific lipid subclasses. A nontargeted metabolomics study on the plasma of sarcopenia patients showed that compared to those in the control group, sarcopenia patients exhibited significant metabolic disorders, and the differential metabolic markers screened were mostly related to lipid and amino acid metabolism disorders [13]. Nevertheless, this study aimed to explore the differences in all metabolites in the plasma of individuals with sarcopenia but lacked a quantitative detection of the population’s lipid profile, making it difficult to accurately evaluate changes in lipid metabolism. Therefore, a relative gap still exists in the research on the relationship between lipid metabolism abnormalities and sarcopenia among populations, which significantly hinders the advancement of mechanistic research. Lipidomics offers a method to investigate this relationship.

Lipids are key components of cells and play an important role in maintaining bodily functions, including participating in energy storage and signal transduction, as well as serving as important bioactive molecules in the human immune system to resist viral and bacterial infections [14,15]. Disorders in lipid metabolism are closely linked to the onset and progression of various diseases [16,17]. Lipidomics employs high-throughput analysis techniques to study the composition and metabolic alterations of lipids in living organisms. Lipid roles in health and disease are examined by analyzing lipid structure and function [18]. Recent lipidomics research has significantly advanced our understanding of disease mechanisms [19,20]. Considering the detrimental effects of abnormal lipid metabolism on muscle strength [6], lipidomics could further explore the occurrence and physiological mechanisms of sarcopenia.

Our research focused on examining the lipid composition and metabolic characteristics of patients with sarcopenia. By analyzing these lipidomic data, we aim to explore the relationship between lipid metabolism abnormalities and sarcopenia among populations at the molecular level, propose possible pathogenic mechanisms and identify potential biomarkers and therapeutic targets that could improve the diagnosis and treatment of sarcopenia.

## 2. Results

### 2.1. Characteristics of the Studied Population

The clinical characteristics of the sarcopenia group and control group, each comprised of 40 individuals, are presented in Table 1. Compared to the control group, the sarcopenia group exhibited a significantly lower BMI, skeletal muscle mass (SMI), calf circumference, and maximum grip strength (*p* < 0.05), with BMI values of 23.70 ± 2.88 and 26.23 ± 2.98, respectively. Additionally, the sarcopenia group had higher levels of high-density lipoprotein cholesterol (HDL-C) (*p* = 0.029). No significant differences were detected between the two groups in terms of marital status, family economic levels, smoking, alcohol consumption, TC, TG, low-density lipoprotein cholesterol (LDL-C), thigh circumference, or daily walking steps.

### 2.2. Univariate and Multivariate Statistical Analysis of Lipids

**Figure 1:**
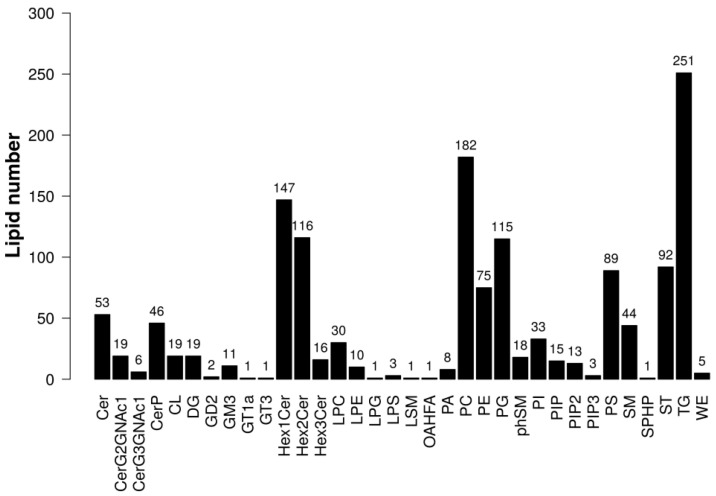
Statistical chart of lipid subclasses and lipid molecule counts. (Note: The horizontal axis represents the detected lipid subclasses, while the vertical axis shows the number of lipid molecules within each subclass).

**Figure 2:**
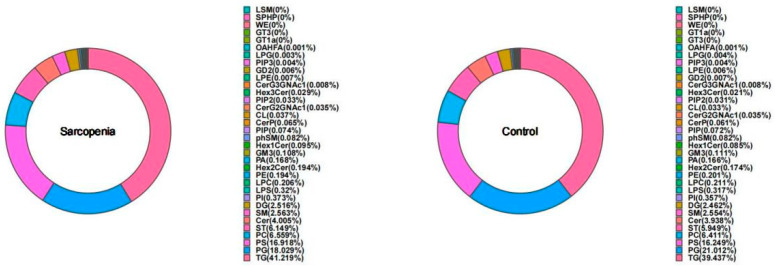
Statistical chart of lipid subclasses and lipid molecule counts.

A total of 34 lipid subclasses and 1446 lipid molecules were detected. The statistical results for the identified lipid subclasses and number of lipid species within each subclass are shown in Figure 1. Notably, the TG subclass contained the highest number of lipid species. The distribution of lipid subclasses within the two groups is depicted in Figure 2, and no significant differences were observed among the subclasses. The top three lipid subclasses in terms of prevalence were TG, phosphatidylglycerol (PG), and phosphatidylserine (PS).

**Figure 3:**
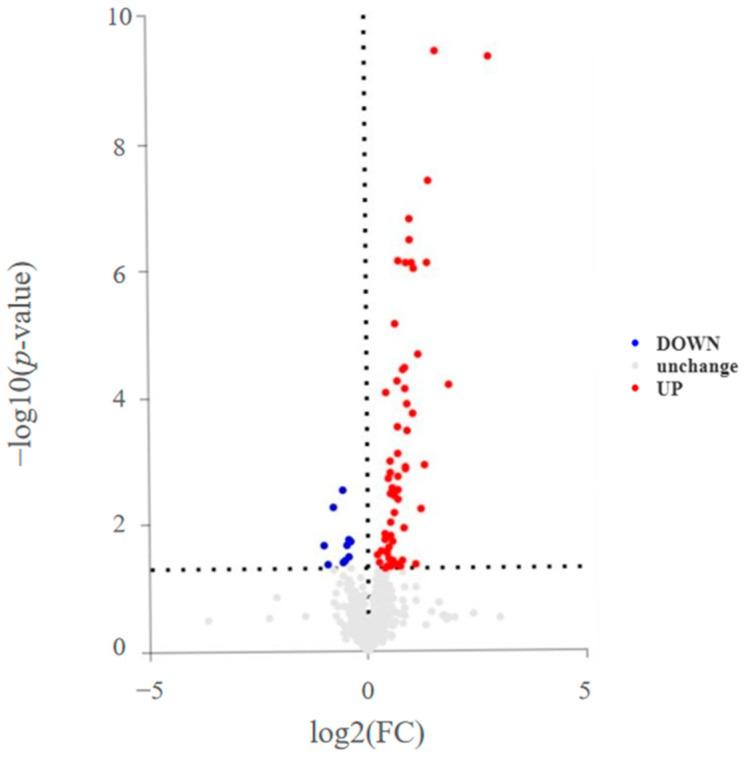
Differential lipid molecules between the two groups—volcano plot. The x-axis in the figure represents the log2-transformed fold change values of differential expression, and the y-axis represents the log10-transformed *p* value. (Note: Compared to the control group, red indicates the upregulation of lipid molecules in the disease group, blue indicates downregulation, and gray represents no significant difference).

The results of the univariate statistical analysis are primarily represented by a volcano plot (Figure 3), where different colors indicate upregulation or downregulation. The results showed that in patients with sarcopenia, the levels of differential lipid molecules were mostly upregulated.

**Figure 4:**
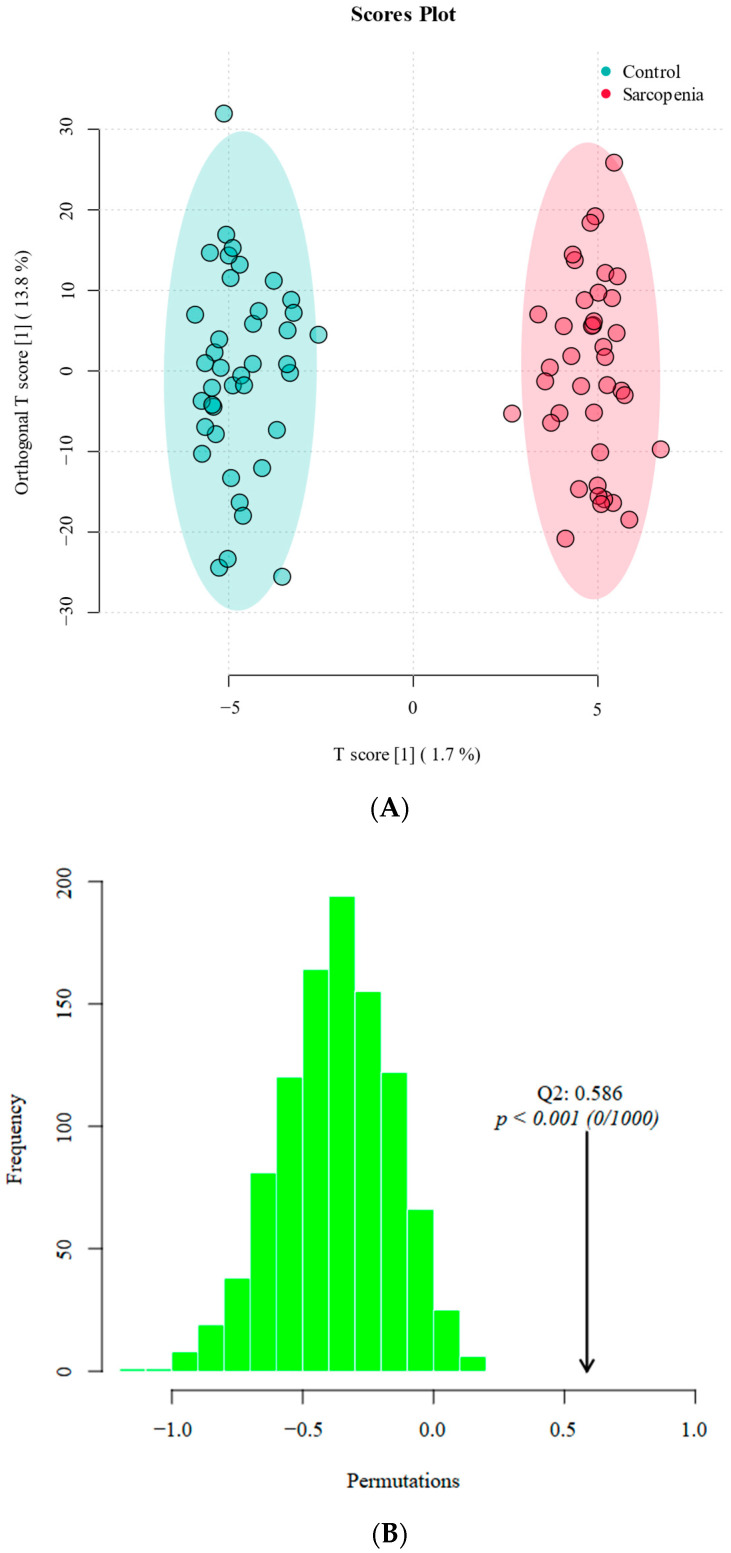
OPLS-DA score plot and permutation test. (**A**) OPLS-DA score plot of the two groups. The *x*-axis and *y*-axis represent the first and second principal components, respectively. Dots of the same color represent various biological replicates within the group. Red represents the sarcopenia group, and green represents the control group. The distribution of dots reflects the degree of difference between and within groups. (**B**) Permutation test of the OPLS-DA model. The *x*-axis represents the permuted Q2 values, and the *y*-axis indicates the frequency of these values.

The results of the orthogonal partial least squares discriminant analysis (OPLS-DA) between the two groups are shown in Figure 4A. The sarcopenia group and control group were well distinguished along the *x*-axis. The model’s R2Y was 0.972, and the Q2 was 0.586, indicating that the model had a good fitting ability and robustness. The permutation test results are presented in Figure 4B. The actual Q2 value of the model (0.586) is marked by an arrow on the right side of the plot. The observed Q2 value was significantly better than those obtained through random permutations, with a *p* value of less than 0.001. This demonstrated that the predictive ability of the model was highly significant and not due to random chance, underscoring the robustness and reliability of the model. Using VIP > 1 and *p* < 0.05 as screening criteria, a total of 80 differential lipid molecules were detected. The differential lipid molecules are presented in Supplementary Table S1.

### 2.3. Differential Lipids between the Sarcopenia and Control Groups

**Figure 5:**
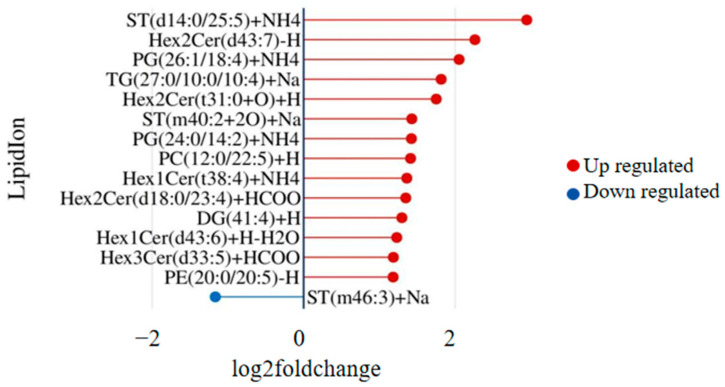
Matchsticks of the 15 most significantly upregulated and downregulated lipids.

Compared to the control group, the sarcopenia group showed an overall upregulation of lipid molecules. Among the 80 differential lipid molecules, including 38 phospholipids, 21 ceramides and glycosylceramides, 20 triglycerides, and 9 other lipids, 53 were upregulated, and 27 were downregulated. Phospholipids were the main molecules experiencing changes. The top 15 differential lipid molecules are shown in Figure 5, with ST(d14:0/25:5) exhibiting the most significant difference between the two groups.

### 2.4. Lipid Coexpression Network Modules Linked to Sarcopenia

**Figure 6:**
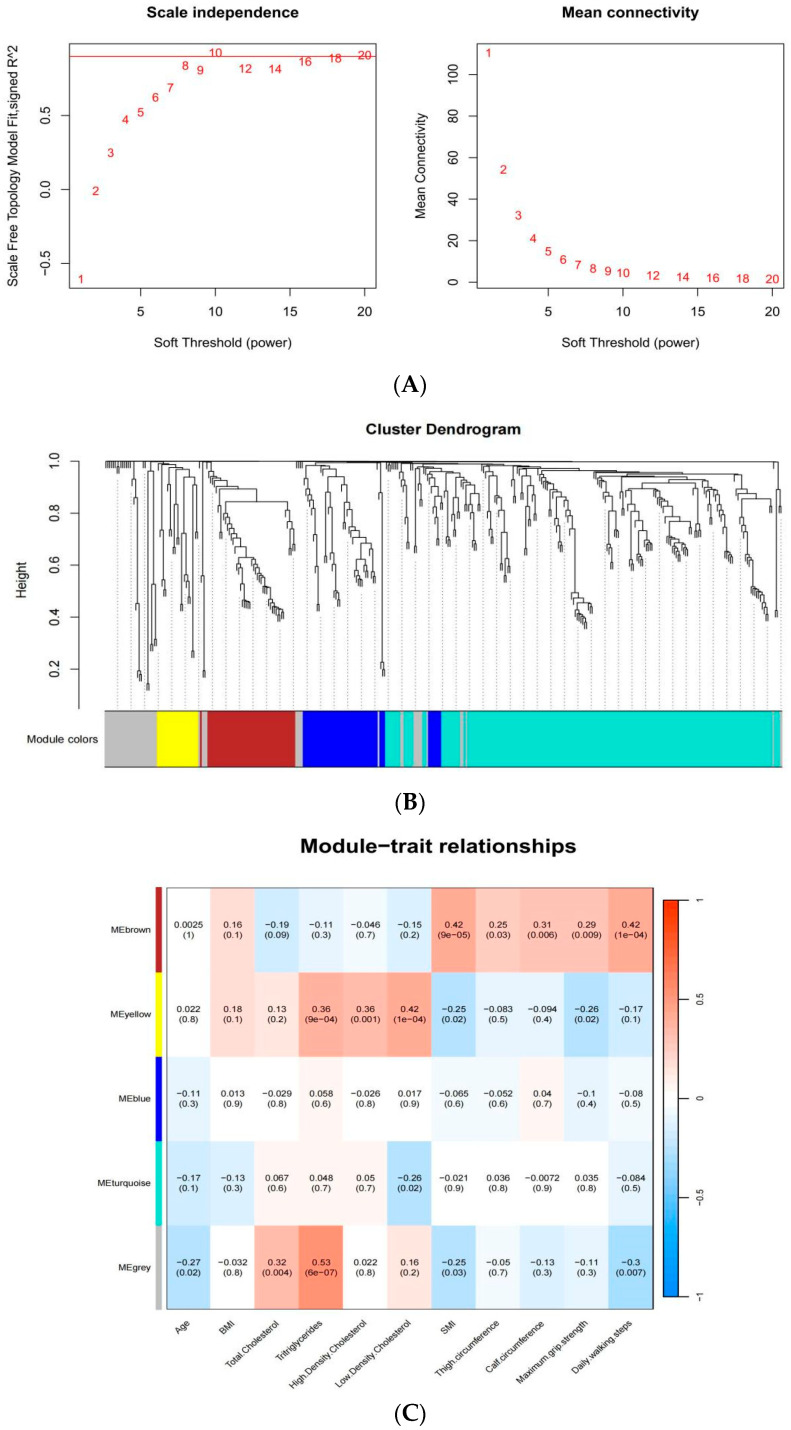
Correlation between lipid metabolism modules and clinical features using WGCNA. (**A**) Determination of optimal soft threshold power for network construction. The red line in Figure 6 (**A**) indicates the threshold for achieving a scale-free topology model fit with RsquaredCut > 0.9, which is considered optimal for network construction. (**B**) A module dendrogram was used to illustrate the lipid distribution across different modules. (**C**) Pearson correlation analysis of the network mod-ules and continuous demographic characteristics of the sarcopenia samples. The color gradient signifies the direction and strength of the correlation, with red indicating positive correlations and blue indicating negative correlations. The numbers inside the boxes represent the correlation coefficients, and the values in parentheses show the corresponding *p* values.

The results of the analysis using a weighted correlation network analysis (WGCNA) are as follows (Figure 6). We included all detected lipid molecules in the WGCNA and set a soft threshold for the WGCNA to construct a scale-free network, ensuring RsquaredCut > 0.9 by setting the soft threshold to 10 (A). Hierarchical clustering based on dissTOM produced a dendrogram identifying five metabolic modules: brown, blue, turquoise, yellow, and gray (B). The brown module consisted entirely of PG lipids; the blue module mainly included sterol (ST), TG, and some PG lipids; and the turquoise module contained the most lipid molecules, including 80 TG, 29 ST, 25 phosphatidylcholine (PC), 22 PS, and 14 sphingomyelin (SM) lipids. The yellow module mainly contained PC, PS, and SM lipids, while the gray module was comprised of the remaining unclassified lipid molecules. The lipids contained in each clustering module are shown in Supplementary Table S2.

The correlation between the network modules and clinical characteristics indicated that the brown module was significantly associated with sarcopenia-related measurements and important clinical traits. Specifically, this module showed a positive correlation with SMI, thigh circumference, calf circumference, maximum grip strength, and daily walking steps (*p* < 0.05), with a stronger correlation with SMI and daily walking steps (R = 0.42). Additionally, the yellow module was positively correlated with triglycerides, high-density lipoprotein, and low-density lipoprotein and negatively correlated with maximum grip strength and SMI, suggesting that this module may influence lipid changes associated with sarcopenia (C).

## 3. Pathway Analysis

**Figure 7:**
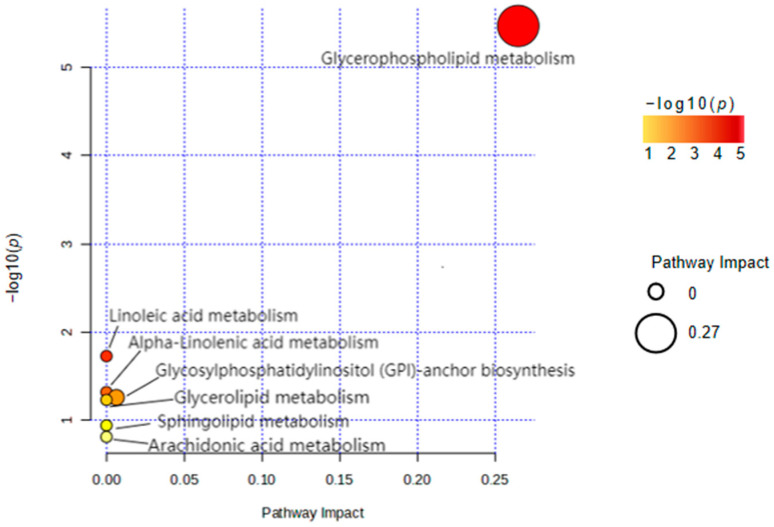
Pathway enrichment analysis of differential lipid molecules (NOTES: The *x*-axis shows the impact score of each pathway. A higher value indicates a greater impact. The *y*-axis shows the statistical significance of the pathway enrichment, with higher values indicating more significant enrichment. The bubble size represents the number of lipid molecules in each pathway. Colors range from yellow to red, with red indicating a higher significance).

MetaboAnalyst identified the most relevant metabolic pathways of differential lipid molecules through internal R script analysis. The results indicated that the glycerophospholipid metabolism pathway exhibited the highest enrichment and statistical significance (Figure 7). This finding suggests that differential lipid molecules between the two groups were predominantly enriched in this pathway, potentially revealing a metabolic pathway that influences disease occurrence.

## 4. Discussion

Sarcopenia has now become a potential public health issue, attracting widespread attention. The causes of sarcopenia are multifaceted, and thus far, no studies have fully unraveled its pathogenic mechanisms. Nevertheless, gaining a deeper understanding of these mechanisms is crucial for effectively diagnosing and treating the condition. Metabolomics provides a reliable tool for further research in this area [21]. Meanwhile, nontargeted lipidomics, an essential branch of lipidomics, allows for the extensive screening and analysis of the detected lipids without predefining specific lipid molecules. Lipidomics systematically examines the lipid profiles and trends in level changes within individuals [22]. This study utilized LC-MS to explore the potential lipid changes associated with sarcopenia, aiming to identify potential biomarkers and pathogenic pathways, thereby offering new insights for the diagnosis and treatment of the disease. We detected a total of 34 lipid subclasses and 1446 lipid molecules, with TG being the subclass containing the highest number of molecules. Eighty differential lipids were identified, most of which were upregulated, with phospholipids being the main lipids exhibiting changes.

Network analysis was employed to identify lipid modules closely associated with the clinical traits of sarcopenia. The brown module, which predominantly consists of unsaturated PG lipids, showed a positive correlation with SMI, thigh circumference, calf circumference, maximum grip strength, and daily walking steps. Conversely, the lipid clusters of PC, PS, and SM in the yellow module showed a positive correlations with TG, HDL-C, and LDL-C levels but negative correlations with SMI and maximum grip strength. A meta-analysis revealed that the OR value of dyslipidemia for the risk of sarcopenia was 1.47, with an overall mean difference of 1.27 for HDL-C and 1.95 for LDL-C, confirming a significant correlation between sarcopenia and dyslipidemia [23]. An interventional trial has indicated that therapy using fish oil-derived n-3 polyunsaturated fatty acids can slow the decline in muscle mass and function typically seen in older adults, suggesting it should be considered a viable treatment option for preventing sarcopenia in this demographic [24]. Additionally, as testosterone is a steroid hormone synthesized from cholesterol, supplementation has been effectively used to enhance muscle mass and function in older men [25]. These studies highlight that fluctuations in lipid levels might alter the risk of developing sarcopenia. Therefore, we speculate that lipid clusters formed by PC, PS, and SM may contribute to the induction of dyslipidemia in patients with sarcopenia. This potential causal link merits further exploration in future research. Previous research has demonstrated that older adults with sarcopenia have distinct skeletal muscle phospholipid profiles. The level of PC is negatively correlated with muscle volume and function [5], which is consistent with our findings. The mitochondrial membrane of skeletal muscle consists of 40% PC, making it susceptible to changes in PC that can trigger mitochondrial dysfunction and impact muscle function [26]. Scholars have indicated that changes in the content of PC may be attributed to the number of unsaturated double bonds. Variations in PC containing unsaturated double bonds are closely associated with the risk of sarcopenia [27]. Additionally, experiments with aging rodent models have confirmed changes in the phospholipids within muscles, which may reflect alterations in muscle cell membrane components during the aging process [28]. PG is composed of glycerol, two fatty acid chains, and a phosphoglyceride group, and its structure allows it to form a bilayer with other lipids in the cell membrane, maintaining the integrity and fluidity of the membrane. Additionally, PG is found in the inner mitochondrial membrane of muscle tissues. Research has revealed that PG possesses potential anti-inflammatory properties, and its immunomodulatory effects depend on the fatty acid composition of PG [29]. Unsaturated PG-liposomes have been found to be more effective in reducing inflammation than their saturated counterparts [30]. This finding supports our results, leading us to speculate that the anti-inflammatory action of PG may slow down the process of muscle tissue atrophy, thereby potentially reducing the risk of sarcopenia. Limited studies have shown a relationship between PG and muscle strength and mass in older adults, providing a basis for a further exploration of their potential connection through co-expression network analysis.

The results of the pathway analysis underscored a potential correlation between glycerophospholipid metabolism and sarcopenia. Consistent with our findings, animal experiments have confirmed that alterations in the glycerophospholipid profile are associated with the phenotype of muscle atrophy in mice [31]. Furthermore, Ferrara et al. demonstrated that skeletal muscle remodeling is associated with an increase in lysophosphatidylcholine acyltransferase 3 (LPCAT3), a key enzyme in glycerophospholipid metabolism. In obese individuals, LPCAT3 can induce fatty acid remodeling in membrane glycerophospholipids, which leads to the inhibition of insulin signaling and glucose tolerance [32]. This research provides substantial theoretical backing for our study and reveals a possible mechanism between skeletal muscle remodeling and glycerophospholipid metabolism.

### Advantages and Disadvantages

Previous studies lacked an exploration of sarcopenia-related lipidomics in human populations. Our research filled this gap by using nontargeted lipidomics to investigate lipid metabolism in individuals with sarcopenia. Consistent with our hypothesis, we found that sarcopenia patients indeed exhibit lipid metabolism disorders. Additionally, our analysis revealed the lipid composition, metabolic changes, phenotype correlations, and potential pathway mechanisms in sarcopenia patients. These findings will contribute to a deeper understanding of the disease and facilitate further research. However, the limitations of this study cannot be ignored. First, our results were based on cross-sectional studies, which prevented us from determining the sequence between differential lipid metabolism abnormalities and sarcopenia, thus limiting causal inference. Future research could benefit from a longitudinal approach, which would allow for tracking changes over time and potentially clarifying the causal pathways involved. Additionally, the small sample size of this study may reduce the likelihood of detecting differential metabolites. However, our objective was to explore potential associations, rather than to establish predictive models; further studies with larger and more diverse populations are necessary to confirm our results and extend their applicability. Particularly, future research should focus on the glycerophospholipid metabolism pathways and the specific lipid clusters observed in this study. Finally, the use of nontargeted lipidomics methods only allowed us to determine the relative values of lipid content changes and lacked the precision and high sensitivity needed for detecting specific molecules. Targeted lipidomics could be essential for future research, to achieve more detailed and accurate insights.

## 5. Materials and Methods

### 5.1. Study Participants

Our study utilized a case-control design and was conducted from March to May 2023. We surveyed 1417 individuals aged 60 and above who were long-term residents of six rural communities in a specific county in Northwest China. In this study, 40 patients with sarcopenia were identified. Each patient was individually matched with one control subject. A case-matched study was performed according to the same sex and a similar age and medical history. Participants who failed to be matched were excluded from the analyses. Basic demographic data, clinical data, physical examination indicators, and questionnaire survey information were systematically collected for all study participants. The exclusion criteria for individuals were as follows: (1) they had a history of psychiatric conditions such as schizophrenia or communication impairments, including cognitive, comprehension, and expressive disorders; (2) were permanently bedridden or had disabilities that interfered with the measurement of the relevant indicators; and (3) were unwilling to participate in the study.

All participants provided written informed consent, and this research obtained approval from the Ethics Committee of Lanzhou University under reference number IRB21010301.

### 5.2. Assessment of Sarcopenia

The SMI was determined using bioelectrical impedance analysis with an InBody 770 (Biospace, Seoul, Republic of Korea). Hand grip strength was evaluated by having participants squeeze a grip strength meter with maximum effort while standing still, and each hand was measured twice, starting with the dominant hand and followed by the nondominant hand, with a one-minute interval between each measurement. The maximum grip strength value of the four measurements was taken as the final record. Gait speed was assessed as participants walked six meters at their natural pace, and the average time of two trials was calculated. The five-time sit-to-stand test involved participants quickly standing and sitting five times from a seated position, with the average time of two trials recorded as the final result.

The diagnosis of sarcopenia was based on the guidelines of the 2019 AWGS [33], with the following criteria: (1) low muscle mass (bioimpedance < 7.0 kg/m^2^ in men and <5.7 kg/m^2^ in women) and (2) low muscle strength (hand grip strength < 28 kg for men and <18 kg for women) and/or low physical performance (6 m walk < 1.0 m/s or 5-time chair stand test ≥ 12 s).

### 5.3. Sample Preparation and Lipid Extraction

All participants were required to fast for at least 8 h during the survey period, after which blood samples were collected in the morning. Serum and plasma samples were quickly separated and stored in a −80 °C freezer until the lipidomic analysis was performed. Lipids were extracted according to the methyl tert-butyl ether (MTBE) method. Two hundred microliters of water and 20 μL of an internal lipid standard mixture was added to the appropriate quantity of the sample and then vortexed. Next, 800 μL of MTBE was added, and the mixture was vortexed thoroughly. Then, 240 μL of prechilled methanol was added, and the mixture was vortexed again. Subsequently, the mixture was sonicated in a cold water bath for 20 min and then allowed to stand at room temperature for 30 min. Afterward, the mixture was centrifuged at 14,000× *g* at 10 °C for 15 min. The upper organic layer was collected, dried under nitrogen, and reconstituted in 200 μL of 90% isopropanol/acetonitrile solution for mass spectrometry analysis. The mixture was vortexed well, a 90 μL aliquot of the solution was centrifuged at 14,000× *g* at 10 °C for 15 min, and the supernatant was analyzed.

### 5.4. LC/MS Method for Lipid Analysis

Chromatographic separation was performed using a UHPLC Nexera LC-30A system equipped with a C18 column, with the column temperature maintained at 45 °C and a flow rate of 300 μL/min. Solvent A was comprised of a mixture of acetonitrile and water in a 6:4 ratio (*v*/*v*) and was enhanced with 0.1% formic acid and 0.1 mM ammonium formate, while solvent B consisted of acetonitrile and isopropanol in a 1:9 ratio (*v*/*v*) and also contained 0.1% formic acid and 0.1 mM ammonium formate. The chromatographic run commenced with 40% solvent B and was held constant for 3.5 min. Solvent B was then incrementally increased to 75% over the next 9.5 min, reaching 99% over the following 6 min, before re-equilibrating to 40% solvent B for the final 5 min.

A mass spectrometric analysis was conducted using a Q Exactive Plus instrument operating in both positive and negative ion modes. The ESI source settings were carefully optimized: the source temperature was maintained at 300 °C, the capillary temperature was 350 °C, the ion spray voltage was set at 3000 V, the S-Lens RF level was set at 50%, and the mass-to-charge (*m*/*z*) scan range was set from 200 to 1800.

The mass-to-charge ratio of lipid molecules and lipid fragments was determined using the following method: 10 fragment maps (MS2 scan, HCD) were collected after each full scan. MS1 had a resolution of 70,000 at M/Z 200, and MS2 had a resolution of 17,500 at M/Z 200.

LipidSearch (Thermo Scientific™, Waltham, MA, USA) was utilized for peak recognition, peak extraction, and the secondary identification of lipid molecules, encompassing more than 30 lipid classes and more than 1,500,000 fragment ions in the database. The parameters used were a precursor tolerance of 5 ppm, a product tolerance of 5 ppm, and a product ion threshold of 5%.

### 5.5. Statistical Analysis

Continuous variables are reported as the mean ± standard deviation (SD), whereas categorical variables are expressed as *n* (%). Demographic characteristics and other factors were compared between the sarcopenia group and the control group using appropriate statistical tests, including analysis of variance, the chi-squared test, Student’s *t* test, and the Kruskal–Wallis H test.

The data analysis included the quantification of lipid statistics, analysis of lipid composition, differential analysis of lipid molecules, clinical correlation analysis, and pathway analysis. Univariate statistical analyses, including a fold change analysis (FC analysis), *t* tests, and nonparametric tests, were employed to identify lipids with significant differences. OPLS-DA, a supervised discriminant statistical approach, was used for a multidimensional statistical analysis, which facilitated the prediction of the sample grouping and the identification of differentially expressed lipids through the establishment of a discriminant model. OPLS-DA has been widely utilized for metabolomics-based differentiation studies [34,35]. Model evaluation parameters R2Y and Q2 were used to assess the stability and reliability of the model fitting [36]. VIP values, along with *p* values, served as screening criteria for identifying differential lipid molecules, specifically, VIP > 1 and *p* < 0.05.

Using WGCNA, we identified metabolic modules highly correlated with sarcopenia and its related clinical traits, aiming to identify potential biological markers [37]. We selected 11 clinical parameters associated with sarcopenia, including blood measurement indicators such as TC, TG, HDL-C, and LDL-C; disease measurement indicators such as SMI, maximum grip strength, and calf circumference; and established traits closely linked to disease risk based on previous literature findings, such as age, daily step count, and thigh circumference. The distance measure employed was 1–TO (dissTOM). We set the minModuleSize to 20, mergeCutHeight to 0.3, and verbose to 3. Additionally, we constructed a scale-free network by setting RsquaredCut > 0.9 and applying a soft threshold of 10. Pearson correlation coefficients were calculated between metabolites within each module feature and various clinical traits, to determine significant clinical modules (*p* < 0.05).

A pathway analysis was conducted using MetaboAnalyst software version 5.0 (https://www.metaboanalyst.ca/ accessed on 17 May 2024) through an internal script in R software, by importing differential lipid data for identification of the most relevant metabolic pathways [38].

## 6. Conclusions

To our knowledge, this study is the first to investigate lipidomics in patients with sarcopenia. We used LC/MS-based nontargeted lipidomics to analyze lipid profiles and found that differential lipid molecules in individuals with sarcopenia were predominantly upregulated. Among the 80 differential lipid molecules, phospholipids accounted for the highest proportion. This finding further confirms the close relationship between sarcopenia and lipid disorders. PG lipids with unsaturated fatty acids showed a positive correlation with the major clinical traits of sarcopenia, such as maximum grip strength and SMI. Conversely, lipid clusters formed by PC, PS, and SM showed a negative correlation, which revealed potential lipid markers. More importantly, we have discovered the potential role of the glycerophospholipid metabolic pathway in the development of sarcopenia. While our study offers some insights into the mechanisms of disease occurrence within populations, its observational design limits our ability to make definitive causal inferences. Future research involving larger sample sizes is crucial. Fundamental animal experiments could provide supporting evidence for our findings, while longitudinal observational studies tracking disease incidence in human populations, along with life course interventions, would be instrumental. Together, these approaches will enhance the validation of our hypotheses and yield more robust evidence for understanding the mechanisms underlying sarcopenia.

## Figures and Tables

**Table 1:** Clinical characteristics of the studied groups (*n* = 80).

	Sarcopenia (*n* = 40)	Control (*n* = 40)	*p*
Age (years)	73.33 ± 3.30	72.88 ± 3.31	0.545
BMI (kg/m^2^)	23.70 ± 2.88	26.23 ± 2.98	**<0.001**
Sex			--
Male	18 (45.0)	18 (45.0)	
Female	22 (55.0)	22 (55.0)	
Marital status			0.999
Married	29 (72.5)	30 (75.0)	
Widowed, divorced, or separated	11 (27.5)	10 (25.0)	
Family economic levels			0.638
Poor	2 (5.0)	3 (7.5)	
Relatively poor	8 (20.0)	11 (27.5)	
Relatively good	24 (60.0)	23 (57.5)	
Good	6 (15.0)	3 (7.5)	
Smoking			0.754
Never	30 (75.0)	32 (80.0)	
Active smoker	5 (12.5)	3 (7.5)	
Former smoker	5 (12.5)	5 (12.5)	
Alcohol consumption			0.797
less than once a month	33 (82.5)	33 (82.5)	
1–3 times per month	2 (5.0)	1 (2.5)	
1–3 times per week	2 (5.0)	2 (5.0)	
More than 3 times per week	1 (2.5)	3 (7.5)	
Stopped drinking	2 (5.0)	1 (2.5)	
TC (mmol/L)	6.70 ± 4.67	5.49 ± 1.41	0.119
TG (mmol/L)	1.54 ± 0.63	1.44 ± 0.90	0.575
HDL-C (mmol/L)	1.62 ± 0.42	1.44 ± 0.30	**0.029**
LDL-C (mmol/L)	3.38 ± 1.05	3.07 ± 0.78	0.135
SMI (kg/m^2^)	5.78 ± 0.71	6.79 ± 0.86	**<0.001**
Thigh circumference (cm)	47.35 ± 8.22	47.95 ± 5.07	0.696
Calf circumference (cm)	31.92 ± 3.41	34.38 ± 3.23	**0.001**
Maximum grip strength (kg)	18.58 ± 5.84	27.30 ± 6.91	**<0.001**
Daily walking steps (steps)			0.246
≤2000	16 (40.0)	8 (20.0)	
2001–4000	6 (15.0)	9 (22.5)	
4001–7000	12 (30.0)	11 (27.5)	
7001–10,000	4 (10.0)	9 (22.5)	
≥10,000	2 (5.0)	3 (7.5)	

Note: Bold values indicate statistically significant differences (*p* < 0.05).

## Data Availability

The datasets used and/or analysed during the current study are available from the corresponding author on reasonable request.

## Supplementary Materials

The following supporting information can be downloaded at: https://www.mdpi.com/article/10.3390/ijms25168793/s1.
